# A Novel LMP1 Antibody Synergizes with Mitomycin C to Inhibit Nasopharyngeal Carcinoma Growth *in Vivo* Through Inducing Apoptosis and Downregulating Vascular Endothelial Growth Factor

**DOI:** 10.3390/ijms13022208

**Published:** 2012-02-17

**Authors:** Yuan Mao, Da-Wei Zhang, Juan Wen, Qing Cao, Ren-Jie Chen, Jin Zhu, Zhen-Qing Feng

**Affiliations:** 1 Department of Otolaryngology—Head and Neck Surgery, The Second Affiliated Hospital of Nanjing Medical University, 121 Jiangjiayuan, Nanjing 210011, China; E-Mails: ymaoent@yahoo.com (Y.M.); chinarenzdw@yahoo.com.cn (D.-W.Z.); wj20091555@163.com (J.W.); ufo-43@163.com (Q.C.); 2 Department of Otolaryngology—Head and Neck Surgery, Jiangsu Province Official Hospital, 65 Jiangsu Road, 210029 Nanjing, China; 3 The Key Laboratory of Cancer Biomarkers, Prevention & Treatment Cancer Center and The Key Laboratory of Antibody Technique of Ministry of Health, Nanjing Medical University, 140 Hanzhong Road, Nanjing 210029, China; E-Mail: fengzhenqing@njmu.edu.cn; 4 Huadong Medical Institute of Biotechniques, 293 Zhongshandong Road, Nanjing 210002, China

**Keywords:** mitomycin C, latent membrane protein 1, antibody targeted therapy, nasopharyngeal carcinoma, apoptosis, VEGF

## Abstract

Combined therapy emerges as an attractive strategy for cancer treatment. The aim of this study was to investigate the inhibitory effects of mitomycin C (MMC) combined with a novel antibody fragment (Fab) targeting latent membrane protein 1 (LMP1) on nasopharyngeal carcinoma (NPC) xenograft nude mice. The inhibitory rates of MMC (2 mg/kg), Fab (4 mg/kg), MMC (2 mg/kg) + Fab (4 mg/kg), and MMC (1 mg/kg) + Fab (4 mg/kg) were 20.1%, 7.3%, 42.5% and 40.5%, respectively. Flow cytometry analysis showed that the apoptotic rate of xenograft tumor cells in the MMC and Fab combination group was 28 ± 4.12%, significantly higher than the MMC (2 mg/kg) group (*P* < 0.01). Immunohistochemical staining showed that VEGF expression in NPC xenografts was significantly inhibited in the combination group compared to the Fab (4 mg/kg) group (*P* < 0.05). In conclusion, both MMC and Fab could inhibit NPC xenograft tumor growth *in vivo* and combination therapy showed apparent synergistic anti-tumor effects, which may be due to the induction of tumor cell apoptosis and the downregulation of VEGF expression. These results suggest that the novel combined therapy utilizing traditional chemotherapeutics and antibody-targeted therapy could be a promising strategy for the treatment of NPC.

## 1. Introduction

Nasopharyngeal carcinoma (NPC) is a kind of malignant tumor that originates from the epithelium of nasopharynx. During the progress of NPC, early cervical lymph node metastasis and distant metastasis may occur, representing a serious problem [1,2]. Current treatments of NPC are mainly radiotherapy and adjuvant chemotherapy, but the total five year survival rate is less than 40% and a series of side-effects are associated with radiotherapy and chemotherapy. Therefore, it is an urgent need to develop effective and safe therapeutics for NPC [3–5].

The infection with the Epstein-Barr virus (EBV) is one of the most important etiologic factors of NPC. EBV is a prototype gamma herpes virus that infects a large number of the population in the world and contributes to the pathogenesis of many EBV-associated cancers, including cervical carcinoma, gastrointestinal carcinoma and NPC [6,7]. Several latent genes are expressed during EBV infection, such as Epstein-Barr nuclear antigen 1 (EBNA1), latent membrane protein 1 (LMP1), LMP2A, and EBV-encoded RNAs (EBERs). LMP1 is a protein with unique characteristics and has been suggested as one of the major oncogenic factors by modulating several pathways involved in NPC, such as vascular endothelial growth factor (VEGF) [8]. Moreover, up to now LMP1 is the only latent protein implicated in the modulation of NPC cell differentiation, transformation and malignancy [9]. Consequently, LMP1 is a promising molecular target for NPC therapy. Although several therapeutic antibodies that target oncogenic products of EBV are currently approved for clinical treatment of NPC [10,11], targeted antibody therapy against LMP1 for NPC treatment has not been reported. In our previous study, we screened a humanized anti-LMP1 antibody Fab from a human naïve Fab phage library but its *in vivo* anti-tumor effect was not characterized [12].

Mitomycin C (MMC) is a classic chemotherapeutics which exhibits effective anti-tumor effects against a variety of solid tumors by inducing apoptosis and reducing drug resistance [13,14]. Notably, the inhibitory effects of MMC against NPC cells have been reported previously [15]. Combination therapy with various drugs is a common strategy in cancer treatment to obtain an additive or synergistic effect and to reduce the potential toxicity. So far, numerous MMC-containing combination remedies have been reported with encouraging clinical effects [16,17]. In this study, we designed a therapy remedy that combined the traditional chemotherapy drug MMC with a novel LMP1 antibody Fab, and evaluated the anti-cancer effects of this new combination therapy in NPC xenograft mice *in vivo*. Our results provided support for further preclinical studies to validate the effectiveness of a combined therapy using MMC and LMP1 Fab for NPC treatment.

## 2. Results and Discussion

### 2.1. MMC in Combination with Anti-LMP1 Fab Exhibits Synergistic Effect to Inhibit HNE2 Tumor Growth *in Vivo*

**Figure 1:**
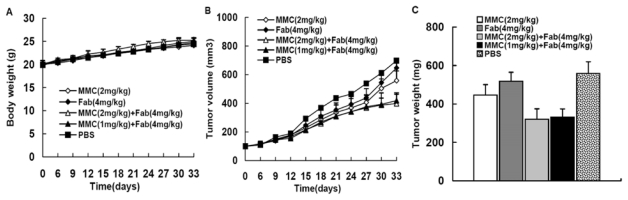
Xenograft nasopharyngeal carcinoma (NPC) tumor growth in five groups. After the inoculation of 5 × 10^6^ HNE2 cells/mL, 40 nude mice were randomly divided into 5 groups and treated as indicated. The body weight (**A**) and tumor volume (**B**) were measured at different time points. After 33 days, all mice were sacrificed and tumors were removed and weighed (**C**).

We established HNE2 xenograft nude mice models that were treated with MMC alone, Fab alone or MMC plus Fab. No animal deaths were observed in the Fab or combination treated groups, but two mice in the MMC treated group died (on day 20 and day 25). As shown in Figure 1, on day 33 the tumor weight and size of PBS control group was 0.558 ± 0.062 g and 697.56 ± 77.48 mm^3^, respectively. However, the tumor weight and size decreased to 0.446 ± 0.054 g and 557.88 ± 67.68 mm^3^ in the MMC (2 mg/kg) treated group, and to 0.517 ± 0.047 g and 646.69 ± 59.18 mm^3^ in the Fab (4 mg/kg) treated group, respectively. In addition, MMC (2 mg/kg) combined with Fab (4 mg/kg) resulted in a further reduction in both tumor weight (0.321 ± 0.054 g) and size (398.67 ± 64.87 mm^3^). Interestingly, when we used decreased concentration of MMC (1 mg/kg) in combination with Fab (4 mg/kg) in xenograft nude mice, the tumor weight (0.332 ± 0.043 g) and size (419.44 ± 53.93 mm^3^) were reduced remarkably compared with the control group and showed no difference in comparison to MMC (2 mg/kg) + Fab (4 mg/kg) group. The inhibition of tumor growth in each treatment group was summarized in Table 1. The results showed that both MMC and Fab showed an inhibitory effect on xenograft NPC tumor growth, but the combination therapy exhibited synergistic effect.

### 2.2. MMC in Combination with Anti-LMP1 Fab Exhibits Synergistic Effect to Induce the Apoptosis of HNE2 Cells *in Vivo*

**Figure 2:**
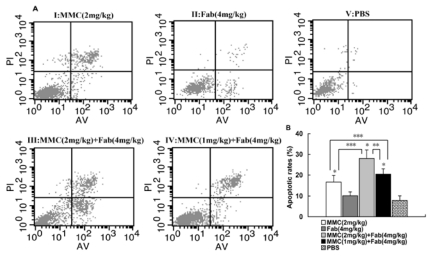
Flow cytometric analysis of the apoptosis of xenograft NPC tumor cells in five groups. After tumor tissues were excised and suspended, tumor cell suspensions were adjusted to a concentration of 1 × 10^6^ cells/mL, and then resuspended in 250 μL of binding buffer, and stained with staining solution containing Annexin V/FITC and PI. The cells were analyzed using a FACScan flow cytometer. A: Representative flow histograms showing the apoptosis of tumor cells. I: MMC (2 mg/kg); II: Fab (4 mg/kg); III: MMC (2 mg/kg) + Fab (4 mg/kg); IV: MMC (1 mg/kg) + Fab (4 mg/kg); V: PBS. B: Comparison of apoptotic rates of xenograft tumor cells in different groups. * *P* < 0.01 *vs.* group V; ** *P* < 0.01 *vs.* Group IV; *** *P* < 0.05 *vs.* group III and IV.

To investigate the potential mechanism by which MMC in combination with anti-LMP1 Fab exhibits synergistic effect to inhibit HNE2 tumor growth *in vivo*, we detected the apoptosis of xenograft tumor cells. Annexin V/PI assay showed that the percentage of apoptotic cells in control group was significantly less than that in MMC (2 mg/kg) treatment group (7.87% *vs.* 16.6%; *P* < 0.01). In combined therapy, the MMC (2 mg/kg) + Fab (4 mg/kg) treatment group showed a higher percentage of apoptotic cells than the control group (28% *vs.* 7.87%; *P* < 0.01) and MMC (2 mg/kg) treatment group (28% *vs.* 16.6%; *P* < 0.01). In addition, in combination therapy with decreased MMC concentration (1 mg/kg) and Fab (4 mg/kg), the percentage of apoptotic cells was still significantly higher than the control group (20.42% *vs.* 7.87%; *P* < 0.01) and MMC (2 mg/kg) group (20.42% *vs.* 16.6%; *P* < 0.05) (Figure 2). These results demonstrate that MMC synergized with anti-LMP1 Fab to induce the apoptosis of HNE2 cells *in vivo*.

### 2.3. MMC in Combination with Anti-LMP1 Fab Exhibits Synergistic Effect to Inhibit VEGF Expression in HNE2 Cells

**Figure 3:**
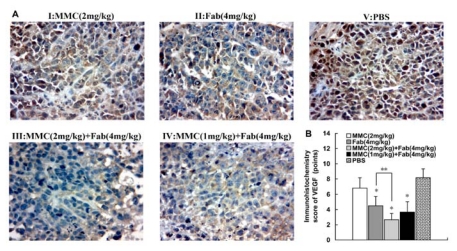
Immunihistochemical staining of vascular endothelial growth factor (VEGF) expression in tumor samples of five groups. A: Representative immunohistochemical staining of VEGF in tumor cells in different groups. Positive staining was observed as brown. I: MMC (2 mg/kg), moderately to strongly positive staining; II: Fab (4 mg/kg), moderately positive staining; III: MMC (2 mg/kg) + Fab (4 mg/kg), weakly positive staining; IV: MMC (1 mg/kg) + Fab (4 mg/kg), weakly to moderately positive staining; B: Comparison of immunohistochemistry score (IHS) of VEGF in xenograft tumor cells in different groups. V: PBS, strongly positive staining. Magnification: 400×. * *P* < 0.01 *vs.* group V; ** *P* < 0.05 *vs.* group III.

Finally we detected VEGF expression in xenografts in nude mice by IHC. Compared with control group, VEGF expression in Fab (4 mg/kg) group, MMC (2 mg/kg) + Fab (4 mg/kg) group and MMC (1 mg/kg) + Fab (4 mg/kg) group was significantly inhibited (*P* < 0.01; Figure 3). In contrast, in MMC (2 mg/kg) group, VEGF expression was not decreased compared with control group (*P* > 0.05). The inhibitory effect on VEGF expression was most significant in the MMC (2 mg/kg) + Fab (4 mg/kg) group but there was no significant difference in VEGF expression between the two combination treatment groups (data not shown).

### 2.4. Discussion

Several cutting-edge treatment strategies have been developed for NPC, including molecular targeted therapy [18], EBV-based immunotherapy [19] and gene therapy [20]. However, no single treatment could achieve a satisfactory therapeutic outcome. Therefore, there is a trend to combine two or more drugs with different mechanisms of action for cancer therapy in clinical protocols. An elaborate strategy of combination therapy may enhance the therapeutic efficacy, decrease the potential toxicity, and minimize or restrain the development of drug resistance [21,22].

In the present study, we observed that MMC and Fab was able to inhibit NPC xenograft tumor growth in a synergistic manner. Moreover, we found no significant difference in anti-tumor effects on tumor volume and weight between two combination therapy groups with different doses of MMC (2 mg/kg *vs.* 1 mg/kg). MMC is known to exhibit toxicity *in vivo* [23]. In this study, no animal death occurred in the Fab or combination treatment groups, while two mice in the MMC group died. Therefore, these results indicate that the lethal toxicity of MMC was reduced due to the combination with Fab. Similar observations were reported earlier on treating breast cancer xenografts with MMC and curcumin [24].

To evaluate the possible mechanism of synergistic anti-tumor effect of MMC and Fab, we performed flow cytometry analysis and found that MMC and Fab combination treatment induced significant higher apoptosis rate of NPC cells resuspected from xenografts compared to single treatment. In our previous *in vitro* study, we reported that MMC induced the apoptosis of NPC cells possibly by activating Caspase-3 [15]. Thus our *in vivo* data are consistent with those of *in vitro* experiments and suggest that MMC in combination with Fab exhibits synergistic effect to inhibit HNE2 tumor growth by inducing apoptosis.

LMP1 is an integral membrane protein which contains three domains: an *N*-terminal cytoplasmic tail, six transmembrane-spanning loops and a *C*-terminal cytoplasmic region. The *C*-terminal region of LMP1 could trigger a variety of signaling pathways such as NF-κB, AP-1 and JAK/STAT to regulate the cell proliferation, immortalization, invasion and metastasis of NPC [7,9]. VEGF is one of the most significant downstream target genes of JAK3/STAT [25] and has been implicated in pathological angiogenesis associated with numerous kinds of tumors [26,27]. Interestingly, VEGF transcription and expression in NPC cell line are enhanced by LMP1 through JAK3/STAT3 pathway [28,29]. Therefore, we proposed that Fab against LMP1 could block the activation of JAK3/STAT3 pathway, thus inhibiting the expression of VEGF in NPC cells. While the elucidation of other mechanisms by which Fab inhibits NPC tumorigenesis is important and will be the focus of our further studies, the present data suggest that it is a feasible strategy to treat NPC by inhibiting LMP1 mediated upregulation of VEGF expression. In our previous study, we isolated Fab fragment against LMP1 from naïve Fab phage library Compared with a full-length antibody, this Fab fragment can be internalized by LMP1 and are suitable for antibody-based immunotherapy [12]. Our results that Fab decreased VEGF expression in NPC xenograft tumors suggest that Fab neutralizes LMP1 and blocks LMP1 mediated downstream signaling. These may help explain the inhibitory effects of Fab alone or together with MMC on NPC growth *in vivo*. Further studies are necessary to elucidate the detailed mechanism by which Fab downregulates VEGF expression in NPC cells and examine whether JAK3/STAT pathway is involved.

## 3. Materials and Methods

### 3.1. Reagents

MMC was purchased from Hisun Pharmaceutical Co., Ltd (Zhejiang, China), dissolved in normal sodium as a 1 mM/L stock solution and stored at 4 °C in the dark. The anti-LMP1 antibody Fab was supplied by Key Laboratory of Ministry of Health, Nanjing Medical University (Jiangsu, China). VEGF antibody was obtained from Sigma (St. Louis, MO, USA). Second antibody was purchased from ZhongShan Goldbridge Co., Ltd (Shanghai, China). Annexin V-FITC apoptosis detection kit was purchased from Biouniquer Technology Co., Ltd (Shanghai, China).

### 3.2. Cell Culture

Human nasopharyngeal carcinoma cells HNE2-LMP1 (LMP1 positive) was purchased from Xiangya Central Laboratory (Hunan, China) and cultured in RPMI-1640 medium (Gibco, San Francisco, CA, USA) supplemented with 10% fetal calf serum (Gibco, San Francisco, CA, USA), penicillin (100 U/mL), and streptomycin (100 μg/mL). The cell culture was maintained at 37 °C with 5% CO_2_ in a humidified atmosphere.

### 3.3. *In Vivo* Tumor Xenograft Model

**Figure 4:**
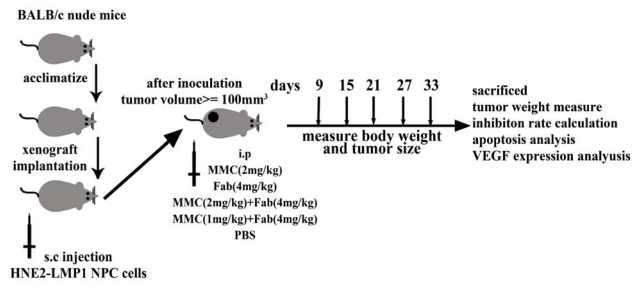
Experimental design of NPC xenograft nude mice model. The mice were s.c. implanted with HNE2-LMP1 cells for about 7 days until tumor volume reached around 100 mm^3^ and then randomly divided into five groups and treated as described in Materials and Methods.

Male 3-week-old BALB/C nude mice with a body weight of approximately 20 g were purchased from SLAC Laboratory Animal Co., Ltd (Shanghai, China), and maintained in standard vinyl cages with air filter tops in a filtered laminar air flow room at 25 °C on a 12 h light/dark cycle; water and food were autoclaved and provided. The experimental design for mice model was shown in Figure 4. For tumor establishment, 5 × 10^6^ HNE2 cells/mL were washed twice with PBS and injected subcutaneously in a volume of 0.1 mL into the flank of mice. After inoculation, tumor-bearing mice were divided randomly into 5 treatment groups (8 mice per group) and treatment initiated when the xenograft solid tumors reached a volume of about 100 mm^3^. Each mouse was injected intraperitoneally on day 1, 4, 7, 10 with different drugs as follows: group I: MMC alone (2 mg/kg); group II: Fab alone (4 mg/kg); group III: MMC (2 mg/kg) + Fab (4 mg/kg); group IV: MMC (1 mg/kg) + Fab (4 mg/kg); and group V: phosphate buffered solution (PBS) as negative control. The animal studies were conducted in accordance with public Health Service policy and approved by the Animal Care and Use Committee of Nanjing Medical University. After xenograft transplantation, mice bearing tumors were observed and tumor size was measured once every 3 days with vernier caliper. The tumor volume in each animal was estimated according to the formula: tumor volume (mm^3^) = *L* × *W*^2^/2 (where *L* was the length and *W* was the width) with the final measurement taken on day 33. At the same time, the body weight of each animal was measured once every 3 days. At the end of the experiments (on day 33), the animals were anaesthetized by CO_2_ and killed. Tumors from each animal were removed, measured and weighed individually. Inhibition rate of tumor growth was calculated by the following formula: Inhibition rate (%) = (tumor weight of control group – tumor weight of experimental group)/tumor weight of control group × 100%. The tumor tissues were also dissected and collected for further examination.

### 3.4. Annexin V/PI Assay for Apoptosis

Tumor tissues from the mice in five groups were excised and suspended, respectively. Apoptosis of tumor cells was assessed by measuring membrane redistribution of phosphatidilserine using an Annexin V-FITC apoptosis detection kit (Biouniquer Technology, Shanghai, China) according to the manufacturer’s protocol. Tumor cell suspensions were prepared and washed with PBS, adjusted to a concentration of 1 × 10^6^ cells/mL, then resuspended in 250 μL of binding buffer and stained with staining solution containing Annexin V/FITC and PI. The cells were analyzed using a FACScan flow cytometer (Becton-Dickinson Immunocytometry System), and the acquired data were analyzed. Additional exposure to propidium iodide (PI) made it possible to distinguish early apoptotic cells (Annexin-positive and PI-negative) from late apoptotic cells (Annexin- and PI- positive). All experiments were performed at least in triplicate.

### 3.5. Immunohistochemistry

The tumors from five groups were excised and then paraffin wax-embedded at Department of Pathology, Nanjing Medical University (Jiangsu, China). Sections (5 μm) were deparaffinized with xylene and then dehydrated in decreasing concentrations of alcohol. Endogenous peroxidase activity was blocked by hydrogen peroxidase (3%) in Tris-buffered saline (TBS) for 30 min. Then the sections were boiled for 10 min in citrate buffer for antigen retrieval. Nonspecific binding was blocked by incubation with 5% goat serum in TBS for 15 min. Next the sections were incubated with VEGF antibody in TBS containing 1% bovine serum albumin at 37 °C for 1 h, then washed with TBS and incubated with EnVision goat anti-mouse/horseradish peroxidase antibody (1:2000) for at 37 °C for 1 h. The color was developed in diaminobenzidine solution and counterstained with Mayer’s hematoxylin. Four fields in each slide were randomly selected and counted, the percentage of positive staining was determined by two clinical pathologists independently using immunohistochemistry score (IHS) [30]. When a conclusion differed, the final decision was made by consensus. IHS was determined by the evaluation of both staining density and intensity. The percentage of positive tumor cells was scored as follows: 1 (0–10% positive cells), 2 (11–50% positive cells), 3 (51–80% positive cells), 4 (81–100% positive cells); and the intensity of staining was scored as follows: 0 (negative), 1 (weakly positive), 2 (moderately positive), and 3 (strongly positive). Multiplication of the intensity and the percentage scores gave rise to the ultimate immunoreactivity score: 0–1 (negative), 2–3 (weak), 4–6 (moderate), and 8–12 (strong).

### 3.6. Statistical Analysis

All data were expressed as mean ± SD and analyzed with SPSS 18.0 statistic software (SPSS Inc, Chicago, IL, USA). Group differences in tumor weight and tumor growth inhibition rate were analyzed by a one-way ANOVA, and post hoc multiple comparison was performed with the LSD method. For all tests, the significance level for statistical analysis was set at *P* < 0.05.

## 4. Conclusions

In conclusion, in this study we combined chemotherapy and antibody targeted therapy to show that MMC and anti-LMP1 Fab combination exhibited synergistic effects to inhibit NPC tumor growth *in vivo* with high efficacy and much less toxicity associated with MMC. The anti-tumor effects appear to be mediated via the induction of apoptosis and the inhibition of VEGF expression. This novel combination therapy represents a promising strategy for the treatment of NPC.

## Figures and Tables

**Table 1:** The inhibitory effects of mitomycin C (MMC) and Fab on NPC xenograft tumor growth *in vivo*. Data were expressed as Mean ± SD (*n* = 6 for group I; *n* = 8 for group II–V).

Treatment groups	Tumor volume (mm^3^)	Tumor weight (g)	Inhibition rate

Dosage (mg/kg)			
I: MMC (2 mg/kg)	557.88 ± 67.68 (*,**,***)	0.446 ± 0.054 (*,**)	20.1%
II: Fab (4 mg/kg)	646.69 ± 59.18 (*,**,***)	0.517 ± 0.047 (*,**,***)	7.3%
III: MMC (2 mg/kg) + Fab (4 mg/kg)	398.67 ± 64.87 (*)	0.321 ± 0.054 (*)	42.5%
IV: MMC (1 mg/kg) + Fab (4 mg/kg)	419.44 ± 53.93 (*)	0.332 ± 0.043 (*)	40.5%
V: PBS	697.56 ± 77.48	0.558 ± 0.062	-

* *P* < 0.001 *vs.* group V;

** *P* < 0.05 *vs.* group III;

*** *P* < 0.05 *vs.* group IV.

## References

[1] Chang E.T., Adami H.O. (2006). The enigmatic epidemiology of nasopharyngeal carcinoma. Cancer Epidemiol. Biomark. Prev.

[2] Guigay J. (2008). Advances in nasopharyngeal carcinoma. Curr. Opin. Oncol.

[3] Tao Q., Chan A.T. (2007). Nasopharyngeal carcinoma: Molecular pathogenesis and therapeutic developments. Expert. Rev. Mol. Med.

[4] Agulnik M., Epstein J.B. (2008). Nasopharyngeal carcinoma: Current management, future directions and dental implications. Oral. Oncol.

[5] Ma B.B., Hui E.P., Chan A.T. (2008). Systemic approach to improving treatment outcome in nasopharyngeal carcinoma: Current and future directions. Cancer Sci.

[6] Thompson M.P., Kurzrock R. (2004). Epstein-barr virus and cancer. Clin. Cancer Res.

[7] Gullo C., Low W.K., Teoh G. (2008). Association of epstein-barr virus with nasopharyngeal carcinoma and current status of development of cancer-derived cell lines. Ann. Acad. Med.

[8] Krishna S.M., James S., Balaram P. (2006). Expression of VEGF as prognosticator in primary nasopharyngeal cancer and its relation to EBV status. Virus Res.

[9] Morris M.A., Dawson C.W., Young L.S. (2009). Role of the Epstein-Barr virus-encoded latent membrane protein-1, LMP1, in the pathogenesis of nasopharyngeal carcinoma. Future Oncol.

[10] Ma B.B., Kam M.K., Leung S.F., Hui E.P., King A.D., Chan S.L., Mo F., Loong H., Yu B.K., Ahuja A., Chan A.T. (2011). A phase II study of concurrent cetuximab-cisplatin and intensity-modulated radiotherapy in locoregionally advanced nasopharyngeal carcinoma. Ann. Oncol.

[11] You B.L., Tourneau C., Chen E.X., Wang L., Jarvi A., Bharadwaj R.R., Kamel-Reid S., Perez-Ordonez B., Mann V., Siu L.L. (2011). A Phase II trial of erlotinib as maintenance treatment after gemcitabine plus platinum-based chemotherapy in patients with recurrent and/or metastatic nasopharyngeal carcinoma. Am. J. Clin. Oncol.

[12] Renjie C., Dawei Z., Yuan M., Jin Z., Hao M., Juan W., Jun M., Qing C., Hong L., Qi T. (2011). A human Fab-based immunoconjugate specific for the LMP1 extracellular domain inhibits nasopharyngeal carcinoma growth *in vitro* and *in vivo*. Mol. Cancer Ther..

[13] Volpato M., Seargent J., Loadman P.M., Phillips R.M. (2005). Formation of DNA interstrand cross-links as a marker of Mitomycin C bioreductive activation and chemosensitivity. Eur. J. Cancer.

[14] Cao Y., Chen D., Zhao P., Liu L., Huang X., Qi C., Liu Y., He H., Wang Q., Liu Y., Chen S. (2011). Intracellular delivery of mitomycin C with targeted polysaccharide conjugates against multidrug resistance. Ann. Biomed. Eng.

[15] Ming H., Zhang D., Lin H., Chen R., Feng Z., Zhu J. (2009). Inhibiting effect of mitomycin C on human nasopharyngeal carcinoma cell line HNE2, HNE2/lmp1 and its mechanism. J. Clin. Med. Prac.

[16] Li M., Zhang J., Wang D., Zhong B., Tucker S., Lu C., Cheng J., Cao C., Xu J., Xu J., Pan H. (2009). A phase II study of intra-arterial chemotherapy of 5-fluorouracil, cisplatin, and mitomycin C for advanced nonresectable gastric cancer. Anticancer.

[17] Xu Y., Kolesar J.M., Schaaf L.J., Drengler R., Duan W., Otterson G., Shapiro C., Kuhn J., Villalona-Calero M.A. (2009). Phase I and pharmacokinetic study of mitomycin C and celecoxib as potential modulators of tumor resistance to irinotecan in patients with solid malignancies. Cancer Chemother. Pharmacol.

[18] Lu Z.X., Ma X.Q., Yang L.F., Wang Z.L., Zeng L., Li Z.J., Li X.N., Tang M., Yi W., Gong J.P. (2008). DNAzymes targeted to EBV-encoded latent membrane protein-1 induce apoptosis and enhance radiosensitivity in nasopharyngeal carcinoma. Cancer Lett.

[19] Louis C.U., Straathof K., Bollard C.M., Gerken C., Huls M.H., Gresik M.V., Wu M.F., Weiss H.L., Gee A.P., Brenner M.K. (2009). Enhancing the *in vivo* expansion of adoptively transferred EBV specific CTL with lymphodepleting CD45 monoclonal antibodies in NPC patients. Blood.

[20] Ho C.H., Chen C.L., Li W.Y., Chen C.J. (2009). Decoy receptor 3, upregulated by Epstein-Barr virus latent membrane protein 1, enhances nasopharyngeal carcinoma cell migration and invasion. Carcinogenesis.

[21] Pallis A.G., Agelaki S., Agelidou A., Varthalitis I., Syrigos K., Kentepozidis N., Pavlakou G., Kotsakis A., Kontopodis E., Georgoulias V (2010). A randomized phase III study of the docetaxel/carboplatin combination *versus* docetaxel single-agent as second line treatment for patients with advanced/metastatic non-small cell lung cancer. BMC Cancer.

[22] Akao Y., Nakagawa Y., Iinuma M., Nozawa Y. (2008). Anti-cancer effects of xanthones from pericarps of mangosteen. Int. J. Mol. Sci.

[23] El-Ghazal R., Podoltsev N., Marks P., Chu E., Saif M.W. (2011). Mitomycin-C-induced thrombotic thrombocytopenic purpura/hemolytic uremic syndrome: Cumulative toxicity of an old drug in a new era. Clin. Colorectal. Cancer.

[24] Zhou Q.M., Zhang H., Lu Y.Y., Wang X.F., Su S.B. (2009). Curcumin reduced the side effects of mitomycin C by inhibiting GRP58-mediated DNA cross-linking in MCF-7 breast cancer xenografts. Cancer Sci.

[25] Niu G., Wright K.L., Huang M., Song L., Haura E., Turkson J., Zhang S., Wang T., Sinibaldi D., Coppola D. (2002). Constitutive Stat3 activity up-regulates VEGF expression and tumor angiogenesis. Oncogene.

[26] Toomey D.P., Murphy J.F., Conlon K.C. (2009). COX-2, VEGF and tumour angiogenesis. Surgeon.

[27] Pircher A., Hilbe W., Heidegger I., Drevs J., Tichelli A., Medinger M. (2011). Biomarkers in tumor angiogenesis and anti-angiogenic therapy. Int. J. Mol. Sci.

[28] Tan Y.N., Tao Y.G., Song X., Tang M., Ai M.D., Cao Y. (2003). Expression of JAK3 in nasopharyngeal carcinoma cell line associated with STAT activation regulated by EB virus encoded protein LMP1. Prog. Biochem. Biophys.

[29] Wang Z., Luo F., Li L., Yang L., Hu D., Ma X., Lu Z., Sun L., Cao Y. (2009). STAT3 activation induced by Epstein-Barr virus latent membrane protein1 causes vascular endothelial growth factor expression and cellular invasiveness via JAK3 And ERK signaling. Eur. J. Cancer.

[30] Friedrich M., Villena-Heinsen C., Reitnauer K., Schmidt W., Tilgen W., Reichrath J. (1999). Malignancies of the uterine corpus and immunoreactivity score of the DNA “mismatch-repair” enzyme human Mut-S-homologon-2. J. Histochem. Cytochem.

